# Biological Activities of Glucosinolate and Its Enzymatic Product in *Moringa oleifera* (Lam.)

**DOI:** 10.3390/ijms26157323

**Published:** 2025-07-29

**Authors:** Jinglin Wang, Saifei Yang, Sijia Shen, Chunxian Ma, Rui Chen

**Affiliations:** Yunnan Key Laboratory of Modern Separation Analysis and Substance Transformation, College of Chemistry and Chemical Engineering, Yunnan Normal University, Kunming 650500, China; 18468042420@163.com (J.W.); feifeiyu0609@163.com (S.Y.); ssj17869164268@163.com (S.S.); 13330431792@163.com (C.M.)

**Keywords:** *Moringa oleifera* Lam., glucosinolate, enzymatic product, bioactivity

## Abstract

In this study, using 70% anhydrous ethanol as the extraction solvent, *Moringa oleifera* Lam. seed powder was extracted with the microwave-assisted extraction method, followed by purification using macroporous adsorbent resin NKA-9. The purified glucosinolate was subsequently hydrolyzed with myrosinase. The glucosinolate and its enzymatic product were identified as 4-(α-L-rhamnopyranosyloxy) benzyl glucosinolate (4-RBMG) and benzyl isothiocyanate (BITC) by UV–Vis, FT-IR, NMR, and MS. The bioactivities, including anti-oxidation, anti-inflammation, and anti-tumor activities of 4-RBMG and BITC, were systematically evaluated and compared. The results show that at 5–20 mg/mL, the anti-oxidation effects of 4-RBMG on DPPH and ABTS free radicals are superior to those of BITC. However, at the same concentrations, BITC has stronger anti-inflammatory and anti-tumor activities compared to 4-RBMG. Notably, at a concentration of 6.25 μmol/L, BITC significantly inhibited NO production with an inhibitory rate of 96.67% without cytotoxicity. Additionally, at a concentration of 40 μmol/L, BITC exhibited excellent inhibitory effects on five tumor cell lines, with the cell inhibitory rates of leukemia HL-60, lung cancer A549, and hepatocellular carcinoma HepG2 exceeding 90%. This study provides some evidence that the enzymatic product, BITC, shows promise as a therapeutic agent for tumor suppression and inflammation reduction.

## 1. Introduction

Glucosinolates are amino acid-derived natural plant products with a thioglucose and a sulfate moiety prevalent in the cruciferous plant family [1,2]. These functional groups endow glucosinolates with numerous bioactivities, including anti-oxidation, anti-tumor, anti-inflammation, and anti-microbial effects [3,4,5]. Additionally, they may aid in alleviating the symptoms of chronic diseases [6]. Glucosinolates are present in most cells, and myrosinase is endogenous to the plant in special myrosin cells. Once the integrity of the plant cell walls is damaged or stressed, glucosinolates and myrosinases will react, allowing release of hydrolysis products including epithionitriles, isothiocyanates, nitriles, oxazolidinethiones, and thiocyanates [7,8,9]. The hydrolysis products of glucosinolates are beneficial for human and animal health, particularly in disease prevention and treatment [10]. For example, isothiocyanates can exert anti-oxidation, anti-inflammation, and anti-tumor effects by regulating cellular signaling pathways and inhibiting the generation of free radicals [3,4,11,12,13]. The studies have further demonstrated in vitro and in vivo the significant bioactivities of glucosinolate and isothiocyanate from *M. oleifera* in hypoglycaemia and anti-tumor applications [14,15,16,17,18].

To our knowledge, while research on the constituents of *M. oleifera* has advanced, systematic studies on the extraction and degradation mechanisms of glucosinolates from *M. oleifera* remain relatively scarce. The microwave-assisted extraction method is one of the novel methods to extract components from plants, which imposes external energy on the plant by microwave [19,20,21]. This method can obtain a higher yield and bioactivity of the active component from the plant at a shorter time and lower solvent and energy consumption than those of the traditional method [22,23,24]. Yan et al. [4] used macroporous anionic resin D301 to purify glucosinolates of *Lepidium meyenii* Walp., which was washed with water and subsequently eluted with potassium nitrate solution. Hebert et al. [25] utilized macroporous anionic exchange resins for the separation of myrosinase and 3-butenyl glucosinolate from the crude extract of mustard seeds in both static and dynamic purification modes. Enzymatic treatments facilitate the acquisition of more specific degradation products, which often show greater efficacy in terms of biological activity in vitro and/or in vivo compared to the original compounds. This study will enhance the potential of *M. oleifera* and its derivatives, rendering them more promising in health.

Based on our previous study and the advantage of the microwave-assisted extraction method, glucosinolate was extracted from *M. oleifera* seed with the microwave-assisted extraction method, purified with macroporous resin, and hydrolyzed with myrosinase. The purified extract and its enzymatic product were determined as 4-RBMG and BITC by UV–Vis, FT-IR, NMR, and MS. Their bioactivities, including anti-oxidation, anti-inflammation, and anti-tumor activities, were evaluated and compared. This study provides some proof that the enzymatic product has the potential as a promising therapeutic agent for suppressing tumors and reducing inflammation.

## 2. Results and Discussion

### 2.1. Glucosinolate and Its Enzymatic Product

**Figure 1:**
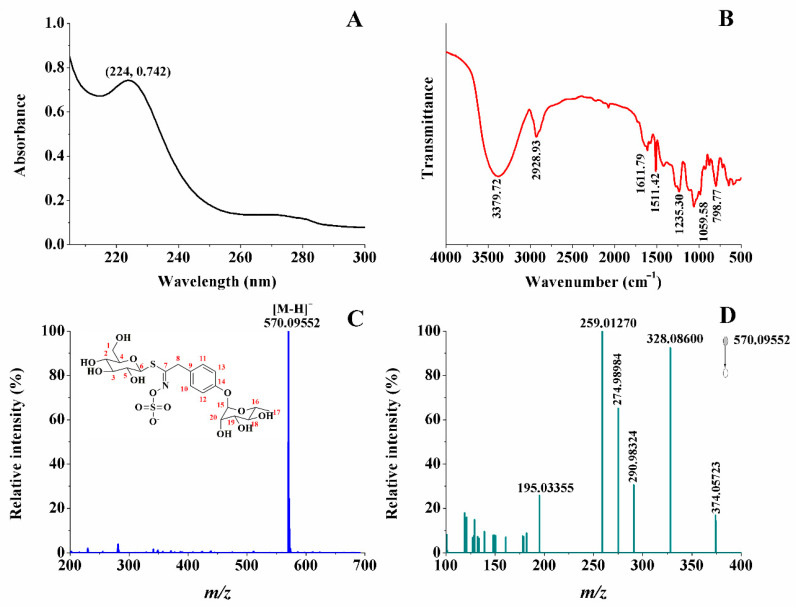
UV–Vis absorption spectrum (**A**), FT-IR absorption spectrum (**B**), mass spectrum (**C**), and MS/MS spectrum (**D**) of the purified glucosinolate, respectively.

The purified glucosinolate has an absorption band around 215–260 nm, and the maximum absorption peak at 224 nm, which is the characteristic absorption peak of S-C=N (Figure 1A) [25]. In Figure 1B, the broad peak at 3379.72 cm^−1^ corresponds to the stretching vibration of hydroxyl group; the peaks at 2928.93, 1611.79, and 1235.30 cm^−1^ correspond to the stretching vibration of the methylene group on side chain and glucopyranose and C=N and C-O on the benzene ring; and the peak at 1059.58 cm^−1^ corresponds to the stretching vibration of C-O on glucopyranose and rhamnose. The peak at 1511.42 cm^−1^ is a characteristic absorption peak of the benzene ring. The combination of peaks at 1511.42 cm^−1^ with the peak at 798.77 cm^−1^ indicates a *p*-disubstitution on the benzene ring. In Figure 1C, a peak at *m*/*z* 570.09552 corresponds to [M − H]^−^. Combined the above results with the NMR data (Figure S1 and Table S1), the chemical structure of purified glucosinolate is determined as 4-(α-L-rhamnopyranosyloxy) benzyl glucosinolate (4-RBMG) [8].

**Figure 2:**
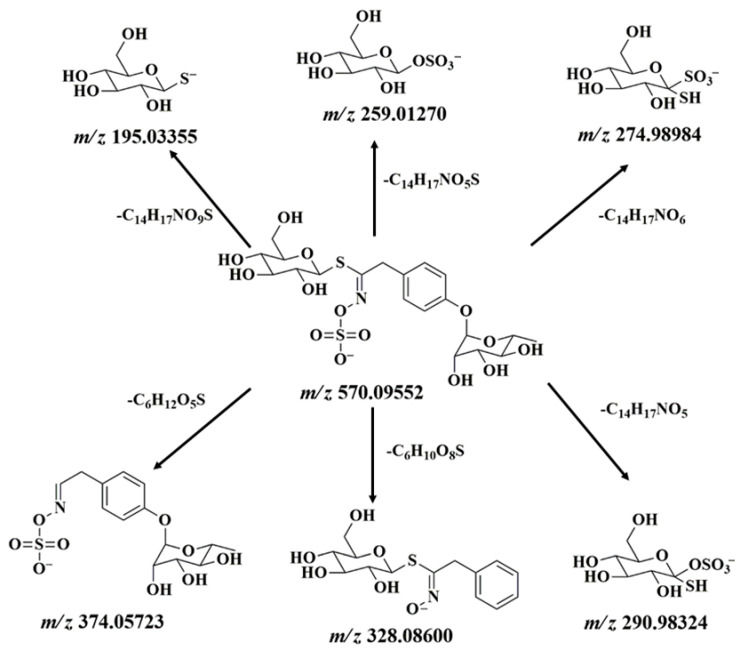
The fragmentation scheme of 4-RBMG in the secondary mass spectrum.

To further determine the structure of purified glucosinolate, the secondary mass spectrum and fragmentation mechanism are given in Figure 1D and Figure 2, respectively. This result further shows that the purified glucosinolate is 4-RBMG.

**Figure 3:**
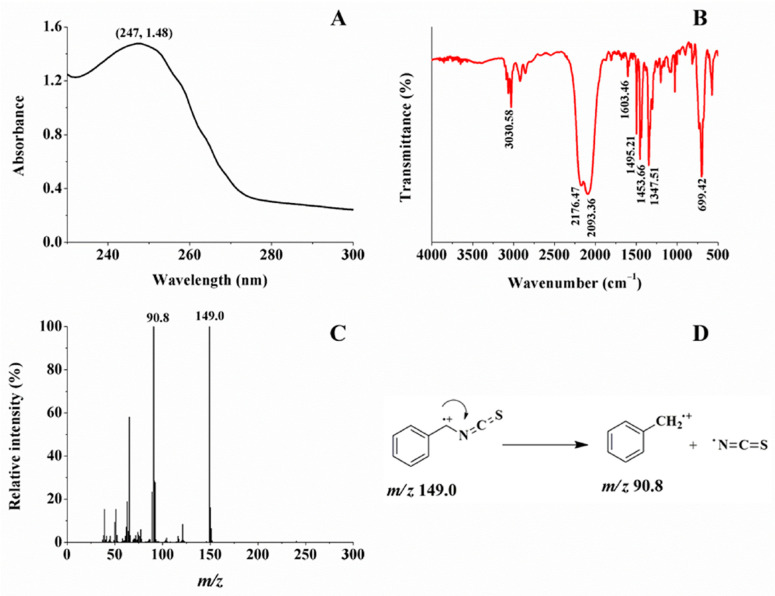
UV–Vis absorption spectrum (**A**), FT-IR absorption spectrum (**B**), mass spectrum (**C**), and fragmentation scheme (**D**) of the enzymatic product, respectively.

In Figure 3A, the enzymatic product has an absorption band around 232–270 nm, and the maximum absorption peak at 248 nm, which is the characteristic absorption peak of N=C=S. In Figure 3B, the peaks at 2176.47 and 2093.36 cm^−1^ are the characteristic absorption peaks of -N=C=S; the peak at 1347.51 cm^−1^ corresponds to the in-plane bending vibration of -CH_2_-; the peaks at 3030.58, 1603.46, 1495.21, and 1453.66 cm^−1^ are the characteristic absorption peaks of the benzene ring. Combined with the above peaks, with the peak at 699.42 cm^−1^, monosubstitution on the benzene ring can further be identified. In Figure 3C, the EI mass spectrum of this product shows that the peak at *m*/*z* 149.0 corresponds to the molecular ion, and the peak at *m*/*z* 90.8 is a major fragment ion obtained through removing an -N=C=S group. Combined with the fragmentation mechanism (Figure 3D) with the ^1^H NMR data (Figure S2 and Table S2) of this enzymatic product, it is identified as benzyl isothiocyanate (BITC) [26].

### 2.2. Biological Activities of 4-RBMG and BITC

#### 2.2.1. Anti-Oxidation Activity

**Figure 4:**
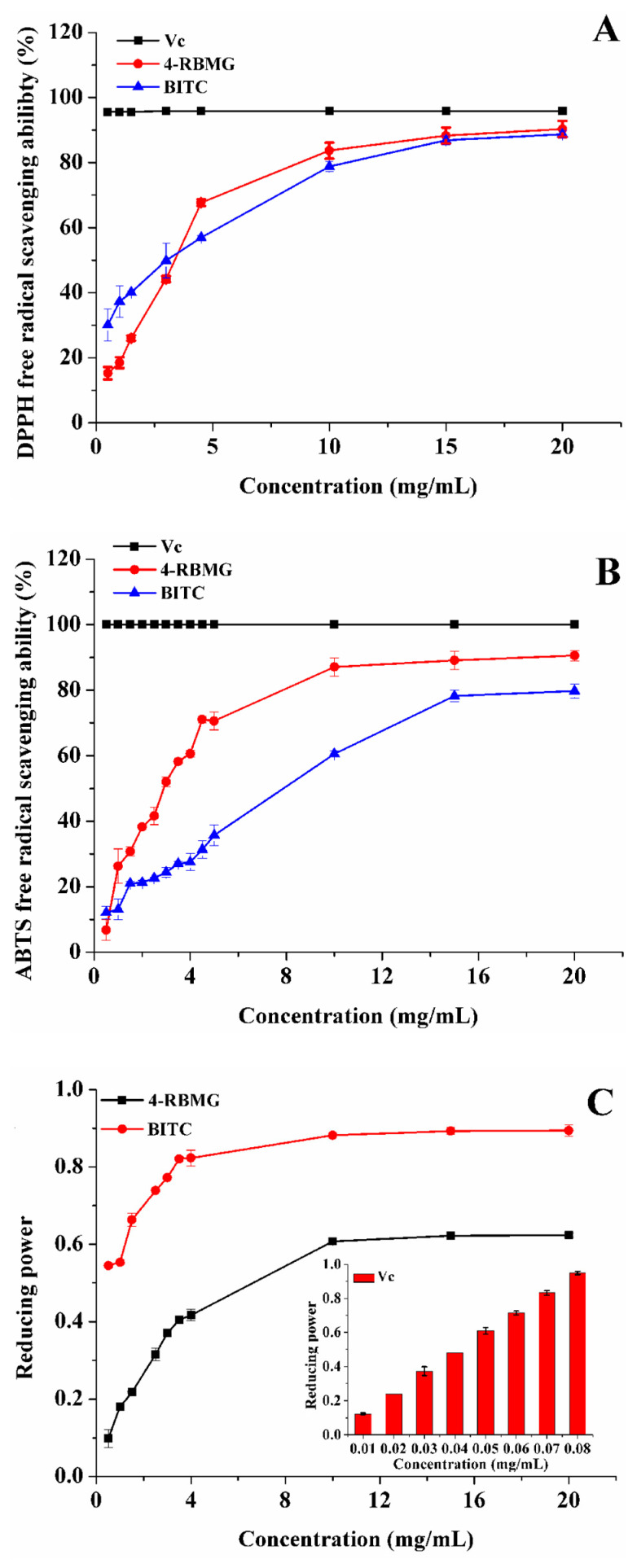
DPPH free radical scavenging capacity (**A**), ABTS free radical scavenging capacity (**B**), and iron reducing power (**C**) of 4-RBMG and BITC with Vc as a positive control, respectively.

In order to investigate the anti-oxidation capacity of 4-RBMG and BITC, the DPPH free radical scavenging capacity was evaluated with vitamin C (Vc) as a positive control. In Figure 4A, the DPPH free radical scavenging abilities of 4-RBMG and BITC gradually increases with increasing sample concentration, eventually reaching a plateau. At a concentration of 10 mg/mL, the DPPH free radical scavenging rate of BITC is 78.81%, slightly inferior to that of 4-RBMG (83.68%). Upon reaching a concentration of 20 mg/mL, the DPPH free radical scavenging rates of BITC and 4-RBMG escalate to 88.72% and 90.32%, respectively.

The ABTS free radical is a stable blue-green group, the color of which is contingent upon the reduction process of the radical. When ABTS reacts with an oxidant to form ABTS^·+^, it becomes light yellow and the absorption peak shifts to 730 nm. In Figure 4B, the ABTS free radical scavenging rates of 4-RBMG and BITC increase with the increase of sample concentration, and ultimately stabilize. At a concentration of 10 mg/mL, the ABTS free radical scavenging rate of BITC is 60.56%, compared to 87.10% for 4-RBMG. When the concentration is increased to 20 mg/mL, the ABTS free radical scavenging rates of BITC and 4-RBMG reach 79.72% and 90.58%, respectively.

The iron-reducing capability serves as an indicator of the anti-oxidant potential of the samples, with the iron-reducing capacity being directly proportional to absorbance. That is, a higher absorbance at 723 nm signifies a more potent iron-reducing capacity of the samples. In this experiment, the iron-reducing capability of 4-RBMG and BITC was assessed with Vc as a positive control. In Figure 4C, the iron-reducing capability of 4-RBMG and BITC progressively increases with the increase of sample concentration, eventually plateauing. The iron reduction capability of BITC is remarkably higher than that of 4-RBMG. For example, at a concentration of 10 mg/mL, the iron reduction capability of BITC is 1.45 times as much as that of 4-RBMG, and akin to that of 0.08 mg/mL Vc.

#### 2.2.2. Anti-Inflammatory Activity

Inflammation is a complex biological process that typically manifests as localized redness, swelling, pain, fever, and dysfunction, reflecting the activated state of the immune system [27,28]. Low concentrations of NO are generally anti-inflammatory and can provide self-protection by inhibiting the over-activation of immune cells and preventing cell damage. Conversely, high concentrations of NO may promote the persistence of inflammation and exacerbate cellular damage. Therefore, the ability to inhibit NO production is a key factor for assessing anti-inflammatory activity.

In Table 1, L-NMMA was used as a positive control to explore the inhibitory effect of 4-RBMG and BITC on NO production. At 50 μmol/L, BITC shows a potent inhibitory effect, with a 99.27% reduction in NO production, significantly outperforming both 4-RBMG (8.22%) and L-NMMA (65.95%). However, at this concentration, BITC was found to be cytotoxic. At 6.25 μmol/L, BITC no longer exhibits cytotoxicity while maintaining a strong inhibitory effect, with a NO production inhibitory rate of 96.67%. The IC_50_ of L-NMMA and BITC are 34.57 ± 0.25 and 1.41 ± 0.12 μmol/L, respectively. This result further confirms that BITC has a stronger ability to inhibit NO production than L-NMMA. The remarkable ability of BITC to inhibit NO production provides valuable support for the development of BITC as an anti-inflammatory pharmaceutical.

#### 2.2.3. Anti-Tumor Activity

**Figure 5:**
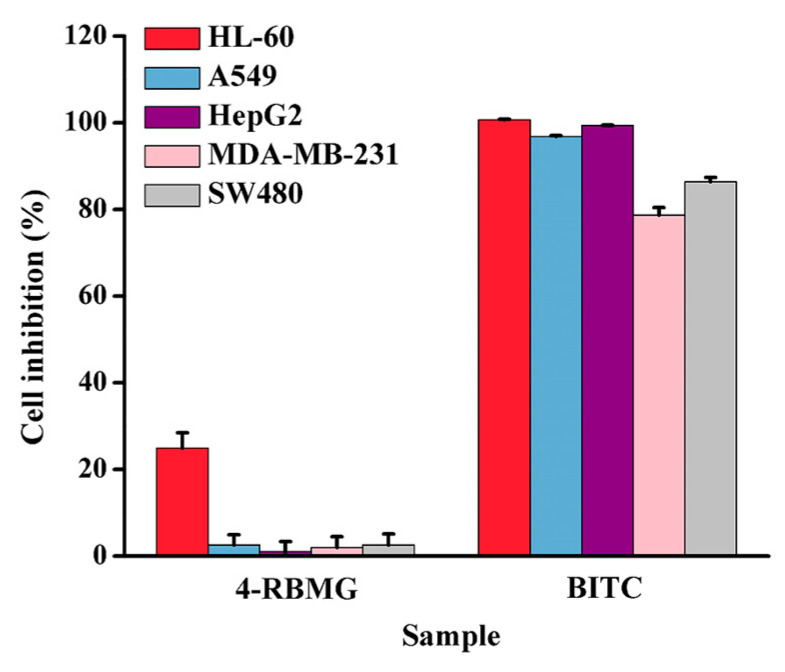
Effect of 4-RBMG and BITC on the inhibitory activity of five tumor cell lines.

Isothiocyanates, including BITC and β-phenethyl isothiocyanates, had inhibitory activities on human breast cancer MCF-7 and mammary epithelial MCF-12A cell lines [7]. Herein, the inhibitory activities of 4-RBMG and BITC on five tumor cells, including leukemia HL-60, lung carcinoma A549, hepatocellular carcinoma HepG2, breast carcinoma MDA-MB-231, and colon carcinoma SW480, were evaluated and compared. The results are shown in Figure 5 and Table S3. At a concentration of 40 μmol/L, the inhibitory effects of BITC on the five strains of tumor cells are significantly better than those of 4-RBMG. In summary, BITC exhibited significant inhibitory effects on five strains of tumor cells, in which the cellular inhibitory rates on leukemia HL-60 (100.68%), lung cancer A549 (96.82%), and hepatocellular carcinoma HepG2 (99.39%) were more than 90%.

Our group isolated benzyl glucosinolate (GTL) from Maca, and its enzymatic product is the same as that of 4-RBMG in this experiment [4]. In order to compare their anti-tumor activities, the inhibitory effects of the two glucosinolates against five tumor cell lines at the same concentration are shown in Table 2. At a concentration of 100 μg/mL, 4-RBMG exhibited a certain inhibitory effect on five tumor cells, and the inhibitory activity is better than that of GTL. For example, due to the different chemical structure, the cell inhibitory rate of 4-RBMG on colon cancer SW480 was 30.95%, which was significantly better than that of GTL (16.64%). This result shows that although the inhibitory activities of 4-RBMG on tumor cell lines, including leukemia HL-60, lung carcinoma A549, and colon carcinoma SW480, are lower than those of BITC, these activities of 4-RBMG are still stronger than those of GTL.

## 3. Materials and Methods

### 3.1. Materials and Reagents

*M. oleifera* was purchased from Fengxi Food Management Co., Ltd. (Kunming, China). ABTS and DPPH were purchased from Shanghai Civic Chemical Technology Co., Ltd. (Shanghai, China). CCl_3_COOH, CF_3_COOH, FeCl_3_, Na_2_HPO_4_·12H_2_O, NaH_2_PO_4_·2H_2_O, K_3_[Fe(CN)_6_], K_2_S_2_O_8_, and Vc were purchased from Tianjin Fengchuan Chemical Reagent Science and Technology Co., Ltd. (Tianjin, China). Macroporous resin NKA-9 was purchased from Beijing Soleberg Griess Reagent Co. (Beijing, China). Lipopolysaccharide (LPS), nitric oxide synthase inhibitor (NOSI), and glucosidase were purchased from Sigma-Aldrich Co., Ltd. (Shanghai, China). Dimethyl sulfoxide (DMSO) was purchased from Biological Industries (Shanghai, China). Paclitaxel and DDP were purchased from Melun Biotechnology Co., Ltd. (Shanghai, China). Five human tumor cancer cells, including leukemia HL-60, lung cancer A549, hepatocellular carcinoma HepG2, breast cancer MDA-MB-231, and colon cancer SW480, were purchased from ATCC (Manassas, VA, USA). RMPI-1640 and DMEM medium were purchased from Biological Industries (Kibbutz Beit-Haemek, Israel). Mouse monocyte macrophage RAW 264.7 was purchased from Shanghai Cell Bank of Chinese Academy of Sciences (Shanghai, China).

### 3.2. Extraction, Purification, and Enzymatic Digestion

A microwave-assisted extraction method was employed using 70% anhydrous ethanol as extraction solvent, with a liquid-to-material ratio of 53:1 (*v*/*m*), under microwave conditions set at 59 °C for 30.5 min. Then, the crude extract of *M. oleifera* was obtained after filtration, evaporation, concentration, and drying.

The crude extract was dissolved in distilled water for further extraction. Initially, it was subjected to extraction three times with an equal volume of petroleum ether, and the aqueous layer was collected. Subsequently, it was extracted with an equal volume of ether three times, again collecting the aqueous layer. Finally, it was extracted three times with an equal volume of water-saturated n-butanol, and the n-butanol layer was collected. This layer was concentrated and dried to obtain the crude glucosinolate. The processed macroporous adsorbent resin NKA-9 was slowly introduced into the chromatography column. The crude glucosinolate, dissolved in distilled water, was pre-adsorbed on the column for 1 h. It was then eluted with distilled water as the eluent, with the flow rate at 1.5 BV/h. The same fractions were collected, concentrated, and dried. This sample was dissolved in methanol and filtered with a 0.22 μm filter membrane, followed by concentration and drying to obtain high-purity glucosinolate.

Glucosinolate and myrosinase (derived from white mustard seeds) were prepared as 50 mg/mL and 1 UN/mL solutions, respectively, in phosphate buffer solution (pH = 7) and stored at −20 °C. A mixture of 2 mL of myrosinase and 1 mL of the sample solution was prepared and placed in a water-bath oscillator to digest at 30 °C and 200 rpm for 2 h. After digestion, the extract was ultrasonically extracted with 5 mL of dichloromethane for 30 min, followed by two additional extractions with 5 mL of dichloromethane. These extracts were filtered, evaporated, and dried at 30 °C to obtain the enzymatic products of glucosinolates.

### 3.3. Characterization of Purified Glucosinolate and Its Enzymatic Product

The UV absorption properties of the purified glucosinolate and its enzymatic product were recorded with a UV-2700i spectroscopy (Shimadzu, Japan) in the wavelength range of 205–300 nm. The chemical structure of these samples was determined by FT-IR absorption spectroscopy (Bruker Daltonics, Bremen, Germany) in the wavenumber range of 4000–400 cm^−1^. These samples were dissolved in deuterated methanol, and the HNMR spectra of the samples were measured using a 500 MHz NMR instrument (Bruker Daltonics, Bremen, Germany). Using methanol as a solvent, purified glucosinolate was scanned with a micrOTOF II time-of-flight mass spectrometer (Bruker Daltonics, Bremen, Germany) in the *m*/*z* range of 100–700 Da in negative ion mode. The experimental parameters of the mass spectrometer were set as follows: flow rate of the sample solution, 3 µL/min; flow rate of nitrogen, 8 L/min; temperature of dry gas, 200 °C; voltage of capillary, 2.6 kV; and voltage of end plate offset, 0.5 kV.

The purified glucosinolate was scanned with a Q Exactive high resolution mass spectrometer (ThermoFisher, Waltham, MA, USA) using methanol as a solvent, the ion source was HESI, and the secondary mass spectra of the samples were scanned by setting the normalized collision energies (NCEs) to 20 eV and 40 eV in the negative ion mode, respectively. The experimental parameters were set as follows: Spray voltage: −3.0 kV; sheath gas flow rate, 40 arb; auxiliary gas flow: 10 arb; heater temperature of auxiliary gas: 350 °C; and ion transfer line temperature: 350 °C.

The enzymatic product was determined by GC–MS (ThermoFisher, Waltham, MA, USA) using a Thermo Scientific TraceGOLD TG-1701MS capillary column (30 m × 0.25 mm × 0.25 µm). The experimental parameters of GC–MS were set as follows: carrier gas, helium; constant flow rate, 1.5 mL/min; temperature of injection, 300 °C; injection volume, 10 µL; split ratio, 20:1; electron impact (EI) ion source; oven temperature, 200 °C; interface temperature, 250 °C; quadrupole temperature, 150 °C; EI energy, 70 eV; and scanning range of *m*/*z*, 35–500 Da.

### 3.4. Biological Activity Assay

The DPPH free radical scavenging assay was performed according to the reported method with some modifications [29]. Solutions of the sample (glucosinolate or its enzymatic product) were prepared with the same concentration of Vc, a positive control. Then, 3.0 mL of 0.05 mg/mL DPPH free radical solution was added to 3.0 mL of sample solutions with different concentrations. These mixtures were allowed to stand at room temperature for 30 min. The absorbances of these samples were measured at 517 nm by UV–Vis absorption spectrometer using ethanol as a blank. The DPPH free radical scavenging rates of the samples were calculated according to the following Equation (1):

(1) $$\mathrm{Scavenging}\;\mathrm{rate}\;(\%)\;=\;\left(1-\frac{A_{1}-A_{2}}{A_{0}}\right)\times 100\%$$

where *A*_0_ is the absorbance of the negative control (DPPH without sample solvent), *A*_1_ is the absorbance of the mixture solution (DPPH and sample), and *A*_2_ is the absorbance of the sample background (sample with ethanol).

An ABTS free radical scavenging assay was performed according to the reported method with some modifications [30]. ABTS standard solution was obtained through mixing 2.45 μmol/mL K_2_S_2_O_8_ solution with 7.00 μmol/mL ABTS solution in a brown reagent bottle and placing it in a dark place overnight (12–14 h). An appropriate amount of ABTS working solution was diluted with distilled water so that the absorbance at 730 nm was around 0.70, and the diluted ABTS working solution was used for subsequent experiments. Then, 0.25 mL of sample (glucosinolate or its enzymatic product) solutions were mixed with 1.5 mL of diluted ABTS free radical stock solution, respectively. The above samples were left for 15 min, and the absorbance of these samples was measured at 730 nm using ethanol as a blank and Vc with the same concentration as a positive control. The ABTS free radical scavenging rates of these samples were calculated according to the following Equation (2):

(2) $$\mathrm{Scavenging}\;\mathrm{rate}\;(\%)\;=\;\left(1-\frac{A_{1}-A_{2}}{A_{0}}\right)\times 100\%$$

where *A*_0_ is the absorbance of the negative control (ABTS without sample solvent), *A*_1_ is the absorbance of the mixture solution (DPPH and sample), and *A*_2_ is the absorbance of the sample background (sample with ethanol).

The determination of iron reducing power was performed according to the reported method with some modifications [31]. Then, 2.5 mL of PBS and 2.5 mL of 1% K_3_[Fe(CN)_6_] solution were mixed with 1 mL of sample solution in a centrifuge tube, heated in a water bath at 50 °C for 20 min, and cooled to room temperature. Prior to centrifugation, 2.5 mL of 10% CCl_3_COOH solution was added to the mixture. Then, the mixture of 2.5 mL of distilled water, 0.5 mL of 0.1% FeCl_3_ solution, and 2.5 mL of supernatant was left to stand for 15 min. The absorbance of the samples was measured at 723 nm.

The anti-proliferation activity assay and anti-inflammatory activity assay were performed according to the reported methods [4].

### 3.5. Statistical Analysis

The statistical analysis of variance was calculated by software SPSS Statistics (Version 27.0, Chicago, IL, USA). Data were subjected to one-way analysis of variance (ANOVA), followed by multiple comparison with least significant differences (LSD) test or Dunnett’s test as appropriate. Statistical significance was considered with *p* <0.05. All the data were expressed as the mean ± SD (*n* = 3).

## 4. Conclusions

Using 70% anhydrous ethanol as extraction solvent, *M. oleifera* seed powder was extracted with the microwave-assisted extraction method and the extract was purified with macroporous adsorbent resin NKA-9. The purified glucosinolate was hydrolyzed with myrosinase. The purified extract and its enzymatic product were determined as 4-RBMG and BITC by UV–Vis, FT-IR, NMR, and MS. The bioactivities including anti-oxidation, anti-inflammation, and anti-tumor activities of 4-RBMG and BITC were evaluated. The results show that the anti-oxidation effects of 4-RBMG and BITC vary with their concentration, and BITC exhibited iron reduction capability at 5–20 mg/mL. At the same concentration, BITC has better anti-inflammatory and anti-tumor activity than that of 4-RBMG. At a concentration of 6.25 μmol/L, BITC has no cytotoxicity and has a strong inhibitory effect on NO generation, with the NO generation inhibitory rate of 96.67%. At a concentration of 40 μmol/L, BITC has obvious inhibitory effects on five tumor cell lines, among which the cell inhibitory rates of leukemia HL-60, lung cancer A549, and hepatocellular carcinoma HepG2 are more than 90%. Additionally, at a concentration of 100 μg/mL, 4-RBMG exhibited a certain inhibitory effect on five tumor cells, and the inhibitory activity was better than that of GTL at the same concentration. The study of isolation, characterization, and biological activity of other glucosinolates is currently in progress in our laboratory.

## Figures and Tables

**Table 1:** Inhibitory rate of L-NMMA, 4-RBMG, and BITC on NO production, respectively.

Sample	Concentration (μmol/L)	Inhibitory Rate of NO Production (%)
L-NMMA	50	65.95 ± 3.63
4-RBMG	50	8.22 ± 2.83
BITC	50	99.27 ± 0.37
	6.25	96.67 ± 0.75

**Table 2:** Inhibitory effect of GTL and 4-RBMG on five tumor cell lines.

Sample	Concentration (μg/mL)	Inhibitory Rate (%)				
		HL-60	A549	SMMC-7721	MCF-7	SW480
GTL	100	2.60 ± 2.15	10.42 ± 0.12	6.36 ± 0.60	18.25 ± 0.63	16.64 ± 1.86
4-RBMG	100	29.44 ± 1.00	15.39 ± 1.34	7.31 ± 1.52	27.57 ± 1.43	30.95 ± 1.16

## Data Availability

Dataset available on request from the authors.

## Supplementary Materials

The following supporting information can be downloaded at https://www.mdpi.com/article/10.3390/ijms26157323/s1.
