# Supplementary material for: Biological Activities of Glucosinolate and Its Enzymatic Product in Moringa oleifera (Lam.)
Source: Int J Mol Sci. 2025 Jul 29;26(15):7323. doi: 10.3390/ijms26157323 (PMC12347210; doi:10.3390/ijms26157323)
Supplement: Supplementary file 1 [file ijms-26-07323-s001.zip › ijms-3724830-supplementary.pdf]

Biological Activities of Glucosinolate and its Enzymatic Product in *Moringa oleifera* (Lam)

Jinglin Wang, Saifei Yang, Sijia Shen, Chunxian Ma and Rui Chen*

Yunnan Key Laboratory of Modern Separation Analysis and Substance Transformation, College of Chemistry and Chemical Engineering, Yunnan Normal University, Kunming 650500, China.

Corresponding author: Dr. Rui Chen 4364@ynnu.edu.cn

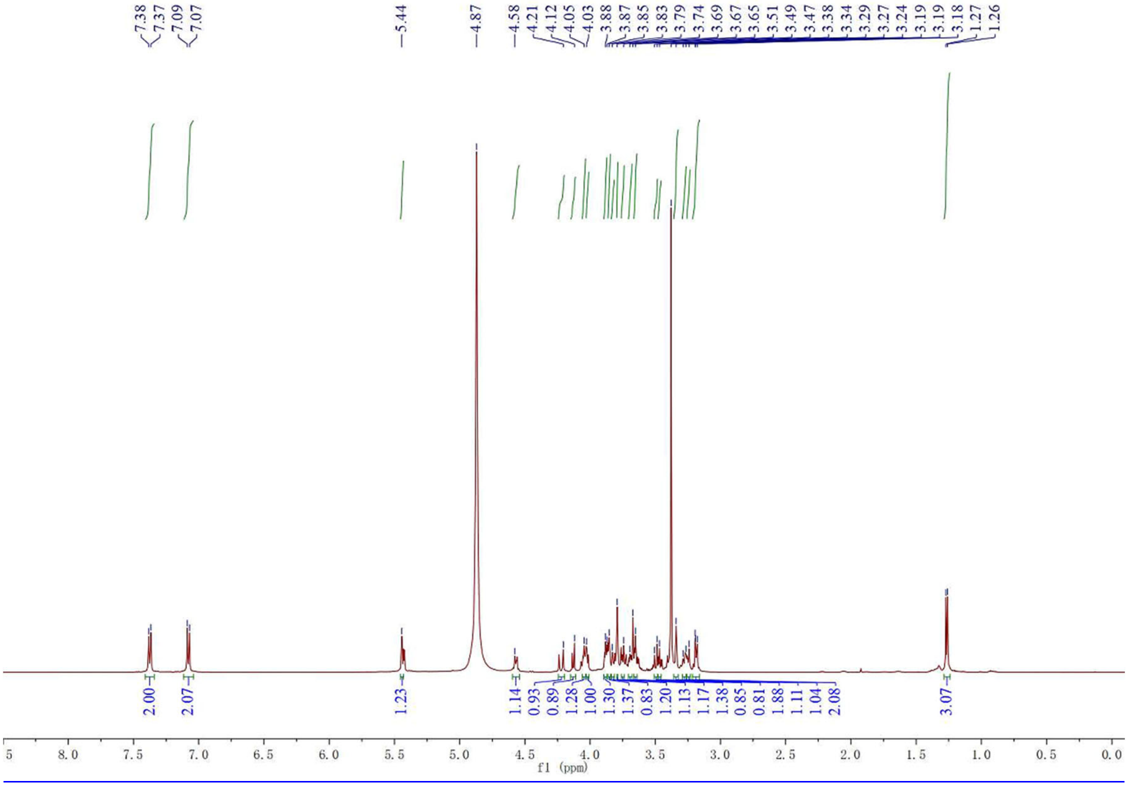

Figure S1 ^{1}H NMR spectra of purified glucosinolate

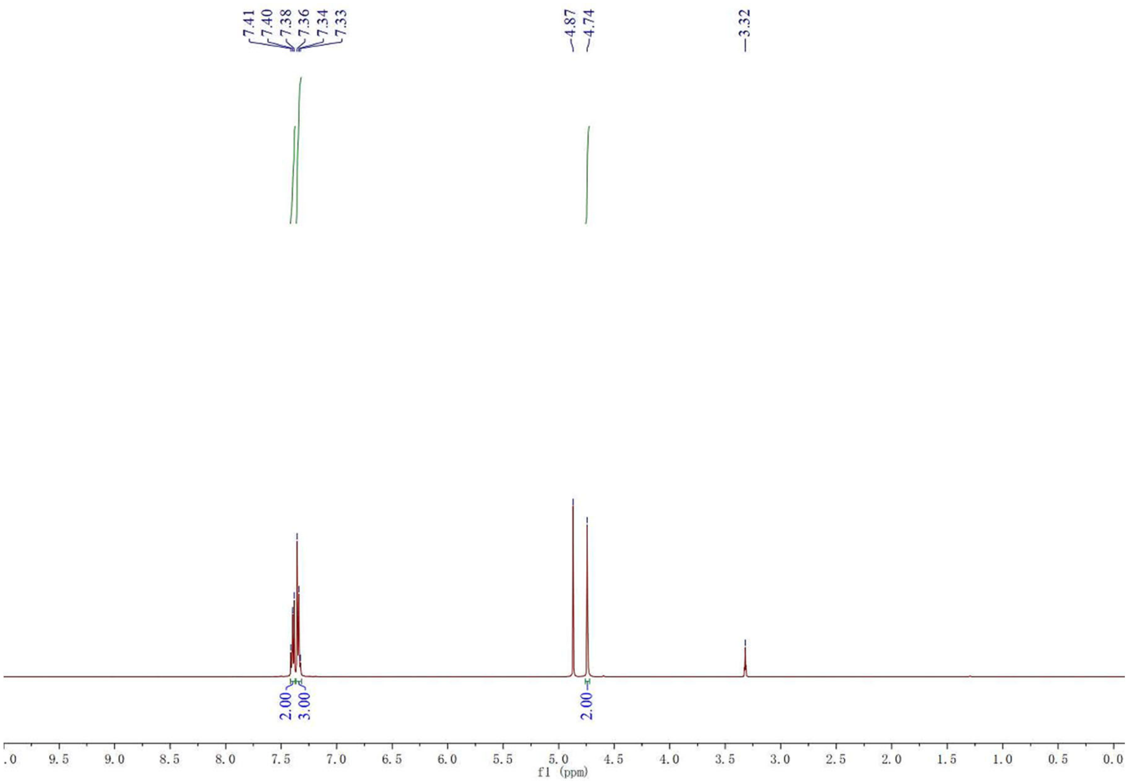

Figure S2 ^{1}H NMR spectra of the enzymatic product

Table S1 ^{1}H NMR data for the purified glucosinolate with coupling constants (J/Hz) in parentheses

No.	δ_H
1	3.12 (t, *J* = 7.5 Hz, 2H)
2	3.19 (m, 1H)
3	3.21 (m, 1H)
4	3.25 (m, 1H)
5	3.17 (m, 1H)
6	4.50 (d, *J* = 9.5 Hz, 1H)
7	-
8	3.27 (s, 2H)
9	-
10, 11	7.31 (d, *J* = 8.5 Hz, 2H)
12, 13	7.01 (d, *J* = 8.5 Hz, 2H)
14	-
15	5.36 (s, 1H)
16	3.62 (m, *J* = 5.6 Hz, 1H)
17	1.20 (d, *J* = 6.2 Hz, 3H)
18	3.42 (m, 1H)
19	3.58 (m, *J* = 12.1, 3.8 Hz, 1H)
20	3.68 (m, *J* = 5.1 Hz, 1H)
1-OH	3.79 (s, 1H)
2-OH	3.81 (s, 1H)
3-OH	3.95 (s, 1H)
5-OH	3.98 (s, 1H)
18-OH	4.05 (s, 1H)
19-OH	4.14 (s, 1H)
20-OH	4.49 (s, 1H)

Table S2 ^{1}H NMR data for the enzymatic product with coupling constants (J/Hz) in parentheses

No.	δ_H
1, 5	7.42-7.37 (m, 2H)
2, 3, 4	7.35 (d, *J* = 7.3 Hz, 3H)
6	-
7	4.74 (s, 2H)

Table S3 Inhibitory effect of 4-RBMG and BITC on five tumor cell lines

Sample	Inhibition rate (%)				
	HL-60	A549	HepG2	MDA-MB-231	SW480
4-RBMG	24.92±3.50	2.59±2.32	1.06±2.30	1.99±2.50	2.57±2.56
BITC	100.68±0.16	96.82±0.22	99.39±0.03	78.66±1.78	86.42±0.97
